# Osteoporosis, Rather Than Sarcopenia, Is the Predominant Musculoskeletal Disease in a Rural South African Community Where Human Immunodeficiency Virus Prevalence Is High: A Cross-Sectional Study

**DOI:** 10.1002/jbmr.4464

**Published:** 2021-11-23

**Authors:** Celia L. Gregson, Tafadzwa Madanhire, Andrea Rehman, Rashida A. Ferrand, Anne R. Cappola, Steven Tollman, Tshepiso Mokoena, The ARK Consortium, Lisa K. Micklesfield, Alisha N. Wade, June Fabian

**Affiliations:** 1 Musculoskeletal Research Unit, Translational Health Sciences, Bristol Medical School, University of Bristol, Bristol, UK; 2 SAMRC/Wits Developmental Pathways for Health Research Unit, Department of Paediatrics, School of Clinical Medicine, University of the Witwatersrand, Johannesburg, South Africa; 3 Biomedical Research and Training Institute, Harare, Zimbabwe; 4 MRC International Statistics and Epidemiology Group, Department of Infectious Disease Epidemiology, Faculty of Epidemiology and Population Health, London School of Hygiene and Tropical Medicine, London, UK; 5 Clinical Research Department, Faculty of Infectious and Tropical Diseases, London School of Hygiene and Tropical Medicine, London, UK; 6 Division of Endocrinology, Diabetes, & Metabolism, Perelman School of Medicine, University of Pennsylvania, Philadelphia, PA, USA; 7 MRC/Wits Rural Public Health and Health Transitions Research Unit (Agincourt), School of Public Health, Faculty of Health Sciences, University of the Witwatersrand, Johannesburg, South Africa; 8 Wits Donald Gordon Medical Centre, School of Clinical Medicine, Faculty of Health Sciences, University of the Witwatersrand, Johannesburg, South Africa

**Keywords:** HIV, BMD, DXA, OSTEOPOROSIS, SARCOPENIA

## Abstract

The rollout of antiretroviral therapy globally has increased life expectancy across Southern Africa, where 20.6 million people now live with human immunodeficiency virus (HIV). We aimed to determine the prevalence of age-related osteoporosis and sarcopenia, and investigate the association between HIV, bone mineral density (BMD), muscle strength and lean mass, and gait speed. A cross-sectional community-based study of individuals aged 20–80 years in rural South Africa collected demographic and clinical data, including HIV status, grip strength, gait speed, body composition, and BMD. Sarcopenia was defined by the European Working Group on Sarcopenia in Older People 2 (EWGSOP2) guidelines, and osteoporosis as BMD *T*-score ≤ −2.5 (if age ≥50 years). The mean ± standard deviation (SD) age of 805 black South African participants was 44.6 ± 14.8 years, 547 (68.2%) were female; 34 (13.2%) were men, and 129 (23.6%) women had HIV, with 88% overall taking anti-retroviral therapy. A femoral neck *T*-score ≤ −2.5, seen in four of 95 (4.2%) men and 39 of 201 (19.4%) women age ≥50 years, was more common in women with than without HIV (13/35 [37.1%] versus 26/166 [15.7%]; *p* = 0.003). Although no participant had confirmed sarcopenia, probable sarcopenia affected more men than women (30/258 [11.6%] versus 24/547 [4.4%]; *p* = .001]. Although appendicular lean mass (ALM)/height^2^ index was lower in both men and women with HIV, there were no differences in grip strength, gait speed, or probable sarcopenia by HIV status. Older age, female sex, lower ALM/height^2^ index, slower gait speed, and HIV infection were all independently associated with lower femoral neck BMD. In conclusion, osteoporosis rather than sarcopenia is the common musculoskeletal disease of aging in rural South Africa; older women with HIV may experience greater bone losses than women without HIV. Findings raise concerns over future fracture risk in Southern Africa, where HIV clinics should consider routine bone health assessment, particularly in aging women.

## Introduction

Life expectancy is increasing more rapidly in sub-Saharan Africa than in any region globally, suggesting that for those who survive early-life challenges, a long old age is now a reality.^(1)^ As populations age, burdens of noncommunicable disease (NCD) are rising, and multimorbidities, including musculoskeletal multimorbidities such as the co-occurrence of osteoporosis and sarcopenia are more common.^(2)^ Yet the natural history of bone and muscle aging in Southern African populations is poorly understood, largely because population-based data are limited. However, recent data suggest vertebral and hip fracture rates are higher among black South African populations than have hitherto been appreciated.^(3,4)^

In Southern Africa, although some risk factors for osteoporosis and sarcopenia including age and female sex may be similar to those in non-African settings, other factors may also be important, such as high prevalence of physical inactivity and human immunodeficiency virus (HIV) infection.^(5)^ Traditional risk factors, such as menopausal-associated bone loss, may behave differently in the context of HIV infection; higher rates of postmenopausal bone loss have been reported in US women living with HIV than those without.^(6)^ HIV is thought to be deleterious to bone through a variety of mechanisms; for example, persistent inflammation, micronutrient and vitamin deficiencies, and the effects of anti-retroviral therapy (ART) itself.^(7,8)^ To date South African studies identifying a negative effect of HIV infection on bone health have been limited to young adults.^(7,9)^

Globally, sarcopenia, only recently recognized as an emerging issue for adults living with HIV,^(10,11)^ is associated with multiple adverse health outcomes including falls, fractures, disability, and death.^(12)^ Within the context of South Africa, lower lean mass has been reported in a small number of perimenopausal women living with HIV,^(13)^ whereas another small study suggested muscle strength may also be affected,^(14)^ although others report no association between HIV and lean mass, muscle strength, or gait speed.^(15)^ Worldwide, sarcopenia definitions vary with the most recent consensus from the European Working Group on Sarcopenia in Older People 2 (EWGSOP2), proposing a structured diagnostic algorithm to aid clinical utility.^(12)^

There are 16 million adults aged ≥40 years currently living in South Africa. Given this age group has an estimated HIV prevalence of 17.2%,^(16)^ it is important to establish the impact of HIV on risk of osteoporosis and sarcopenia. We aimed to determine the prevalence of osteoporosis and sarcopenia; to understand the associations between age and bone mineral density (BMD) in both men and women and establish whether these associations differ according to HIV infection; and to explore the relationships between sarcopenia and its components (grip strength, lean mass, and gait speed) and BMD.

## Subjects and Methods

### Study population

This cross-sectional study was conducted in the Medical Research Council/Wits University Rural Public Health and Health Transitions Research Unit (Agincourt) in Bushbuckridge, a rural subdistrict of the Mpumalanga province, in northeastern South Africa.^(17)^ The Agincourt Health and Socio-Demographic Surveillance System (HDSS) site (90,000 people), established in 1992, includes 31 research villages. Annual updates of vital information on residents have facilitated a longitudinal platform supporting observational and interventional work along the life course involving, among others, individual and population health, migration, and urbanization.^(17–19)^ Municipal infrastructure is limited, with inadequate access to water and electricity, and unemployment rates are high, leading to high rates of labor migration. Health provision is managed through a primary healthcare system consisting of six clinics, two health centers, and three district hospitals. This study of bone and muscle was nested within a wider study of multimorbidity set up to study renal disease.^(20)^

Potential participants were selected from the residents of the HDSS who form a community-based open cohort of inhabitants of 25 neighboring villages. Participants who agreed to take part were visited at home (November 2017 to November 2018), when questionnaires were administered, anthropometry measured, and point of care tests performed to determine HIV status and blood glucose levels. A total of 2759 adults aged 20–80 years, using a 1 female: 1 male ratio, were invited to participate, of whom 2021 (80.1%) consented (phase 1). In phase 2, households were re-visited to offer half the participants a referral date to attend the clinic for assessments, including BMD and sarcopenia. Participants who had acute febrile illness, uncontrolled seizures, or severely elevated blood pressure, and women who were pregnant or breast-feeding were excluded. In total 933 individuals (46.2%) attended for further assessments, from whom we were able to ascertain dual-energy X-ray absorptiometry (DXA) and muscle measures and an HIV status in 665 of 2021 (32.4%). We supplemented this population by further word-of-mouth recruitment within the same community (phase 3). Of 245 who volunteered, 140 (57%) had a DXA and muscle measures performed, and their HIV status established (Supplemental Fig. S1).

### DXA

BMD and body composition were assessed using a Hologic Discovery A DXA scanner (analyzed using version 13.5 software) (Hologic Inc., Bedford, MA, USA), measuring lumbar spine (LS) (L_1_–L_4_), total hip (TH), femoral neck (FN), and total body less head (TB-LH) BMD (the head was excluded due to potential artifact from hair accessory use in women), together with TB-LH fat-free soft tissue mass (as a proxy for lean mass) and fat mass. Appendicular lean mass (ALM) was calculated as the sum of the fat-free soft tissue mass in both upper and lower limbs. All DXAs were performed by one trained radiographer, with coefficients of variation for BMD of 0.94% for TB and 1.08% for TH. BMD *Z*-scores for those age <50 years and *T*-scores for those ≥50 years were derived using white sex-specific reference data from the National Health and Nutrition Examination Survey (NHANES) III,^(21)^ as recommended by the International Society for Clinical Densitometry (ISCD) for African populations in the absence of country (or region)-specific reference data.^(22)^

### Assessment of sarcopenia

Upper limb grip strength was measured as the highest of three right-handed and three left-handed measures of hand dynamometry using a Baseline Digital Smedley Spring Hand Dynamometer (Fabrication Enterprises Inc., White Plains, NY, USA).^(23)^ Maximal gait speed (meters per second) over 6 m was measured because it declines more steeply between 20 and 80 years than normal walk speed^(24)^: after a practice walk, they were asked to “walk as fast as you can” between two lines on the floor, with the mean of three measurements taken. Because there is no consensus on the definition of sarcopenia in African populations, we followed the most recent recommended approach proposed by the European Working Group on Sarcopenia in Older People 2 (EWGSOP2) to define a three-category variable^(12)^: (i) probable sarcopenia based on low muscle strength (grip strength <16 kg for women, <27 kg for men); (ii) confirmed sarcopenia when, in addition, low muscle quantity was confirmed by a lean mass index (ALM/height^2^ index <5.5 kg/m^2^ for women, <7.0 kg/m^2^ for men); (iii) severe sarcopenia when low physical performance was also evident using gait speed (gait speed ≤0.8 m/s).^(12)^

### Demographic and clinical variables

We collected data on demographic factors including age, sex, place of residence, tobacco smoking, alcohol use, and gravidity. Women were asked if they considered themselves to still be “of child-bearing potential.” Medical history and treatment for chronic diseases such as HIV and diabetes mellitus were self-reported using the World Health Organization (WHO) STEPwise Approach to NCD Risk Factor Surveillance (STEPS).^(25)^ Socioeconomic status (SES) was derived using methodology validated within this population (based on housing type and household assets) and participants were assigned to SES quartiles.^(26)^ We estimated physical activity using the active transport domain (eg, walking and cycling) of the Global Physical Activity Questionnaire.^(27,28)^ We measured height and weight, and calculated body mass index (BMI) as weight/height^2^ (kg/m^2^): underweight (<18.5 kg/m^2^), normal weight (18.5–24.99 kg/m^2^), overweight (25.0–29.99 kg/m^2^), and obese (>30.0 kg/m^2^).

### HIV testing

Those who reported being HIV positive had their ART history recorded. If they had not been tested or reported being HIV negative, HIV testing and counseling was offered with testing conducted according to 2015 National Guidelines, which included onward referral to HIV services for those newly diagnosed and/or not linked to care.^(29)^ Those with self-reported HIV and those with a confirmed HIV status were included in analyses.

### Ethical considerations

Ethical approval was granted by the University of the Witwatersrand Human Research Ethics Committee (#M160938) and the Mpumalanga Research and Ethics Committee. Written informed consent, in the participant’s first language (primarily Shangaan) was obtained from participants.

### Statistical analyses

Data were managed in Research Electronic Data Capture (REDCap; https://projectredcap.org/) and exported to Stata 15 (StataCorp, College Station, TX, USA) for analyses.^(30,31)^ All quantitative variables were assessed for normality and outliers. Normally distributed variables are presented as mean ± standard deviation (SD), whereas non-normally distributed variables are presented as median with an interquartile range (IQR). Categorical variables are presented as frequencies and proportions. Comparisons of continuous variables between men and women, and between those with and without HIV, were performed using the Student’s *t* test and the Mann-Whitney *U* or Kruskal-Wallis test for normally and non-normally distributed variables, respectively, whereas the chi-square test was used to compare categorical variables.

Linear regression was used to determine the association between age and BMD, stratified by sex, HIV status, and by age group (<50 years or ≥50 years), then linear regression betas were used in a piecewise regression model to maximize precision (generating standard errors for the beta), while taking account of any non-constant relationship between age and BMD in the two age strata. Multivariable linear regression was used to determine the factors independently associated with BMD. An initial simple model adjusted for age and sex. Then the individual components of sarcopenia (grip strength, ALM/height^2^, gait speed), plus fat mass (given the high prevalence of obesity in South Africa) and HIV status were added to determine the independent predictors of BMD. Fat mass was used in preference to body weight to avoid over adjustment of ALM/height^2^. The correlations between the individual components of sarcopenia were checked, and variance inflation factors (VIFs) were checked after fitting the models to avoid colinearity. Logistic regression was used to determine the association between the presence of osteoporosis and sarcopenia. To determine whether women with HIV, age ≥50 years (as a proxy for menopause) had evidence of altered BMD, compared with women without HIV, we used our fully adjusted model (age, grip strength, ALM/height^2^, gait speed, and fat mass) and tested whether the association between age (<50 years or ≥50 years) and BMD outcomes differed by HIV status using the likelihood ratio test.

## Results

### Characteristics of study participants

The 805 study participants with complete DXA data and an established HIV status were more often female, overweight/obese, of higher SES, and were marginally older than those who were invited but did not have complete data (Supplemental Table S1). Four individuals who lacked grip strength and 36 lacking gait speeds were similar to those with complete muscle measures (Supplemental Table S2). The 547 women and 258 men studied were of similar age (mean ± SD, 44.8 ± 14.2 and 44.1 ± 16.1 years, respectively); however, men were more likely to drink alcohol, smoke tobacco, and have lower SES than women (Table 1). All participants identified as black South African. Among women, 34.2% self-reported no longer being of child-bearing potential. Only 27 (6.1%) women were nulliparous, whereas 194 (61.8%) had had three or more children. Seventeen (7.5%) men and 39 (8.2%) women had diabetes mellitus (self-reported and/or random plasma glucose ≥11.1 mmol/l). No person reported taking oral steroids, nor did any woman report use of estrogen replacement therapy. Men spent more time walking and/or cycling than women. Although men were taller than women, women were heavier with substantially higher BMI; 73.1% of women were classified as overweight or obese compared to 43% men (Table 1).

In total, 34 (13.2%) men and 129 (23.6%) women were living with HIV (146 self-reported an established diagnosis and 17 tested positive), of whom 143 (88%) were taking ART. Both women and men with HIV were less likely to be obese than those without HIV (Table 1).

### BMD and the prevalence of osteoporosis in those with and without HIV

Women had lower measured BMD at all four skeletal sites compared with men, with lower TB-LH and LS *Z*-scores, with no marked sex differences seen in TH or FN *Z*-scores (Table 2). Women with HIV had lower absolute BMD, and substantially lower BMD *Z*-scores at all skeletal sites compared with those without HIV, particularly at the FN (mean *Z*-score difference 0.47; 95% confidence interval [CI], 0.25–0.69). Similarly, men with HIV had lower absolute FN BMD and BMD *Z*-score than those without HIV (mean *Z*-score difference 0.49; 95% CI, 0.06–0.92), with differences at non-hip sites being less pronounced.

Among those age ≥50 years (n = 296) the prevalence of osteoporosis (*T*-score ≤ −2.5) in women was 18.4% (95% CI, 13.3–24.5) at the LS, 19.4% (95% CI, 14.2–25.6) at the FN, and 5.0% (95% CI, 2.4–9.0) at the TH, whereas in men prevalence was 8.4% (95% CI, 3.7–15.9), 4.2% (95% CI, 1.2–10.4), and 3.2% (95% CI, 0.7–9.0), respectively (Table 2). In women, osteoporosis was more common at all skeletal sites in those with HIV (37% [95% CI, 21.5–55.1] of women with HIV age ≥50 years had osteoporosis at the FN compared to 15.7% [95% CI, 10.5–22.1] in women without HIV); in men HIV was not associated with the presence of osteoporosis, although numbers were small.

### Prevalence of sarcopenia in men and women with and without HIV

Within the study population overall, the three sarcopenia measures were all positively correlated with one another, although not strongly: gait speed versus ALM/height^2^ *r* = 0.16, ALM/height^2^ versus grip strength *r* = 0.39, and grip strength versus gait speed *r* = 0.43. In both men and women, lean mass and ALM were lower in those living with HIV than without, whereas grip strength and gait speed were similar. However, only 19 men and five women were classified as having low ALM/height^2^. Hence, overall, 11.6% (95% CI, 8.0–16.2) men and 4.4% (95% CI, 2.8–6.5) women were found to have probable sarcopenia, with no individual having confirmed or severe sarcopenia. Probable sarcopenia was not associated with HIV infection. Notably, fat mass was markedly lower in those with HIV infection.

Among all men and women aged 50 years and older, osteoporosis at the TH was associated with higher odds of probable sarcopenia (odds ratio [OR] 5.6; 95% CI, 1.54–20.3; *p* = 0.009); however, this association was not seen at the other skeletal sites (Supplemental Table S3).

### Age associations with BMD in those with and without HIV

**Fig. 1.**
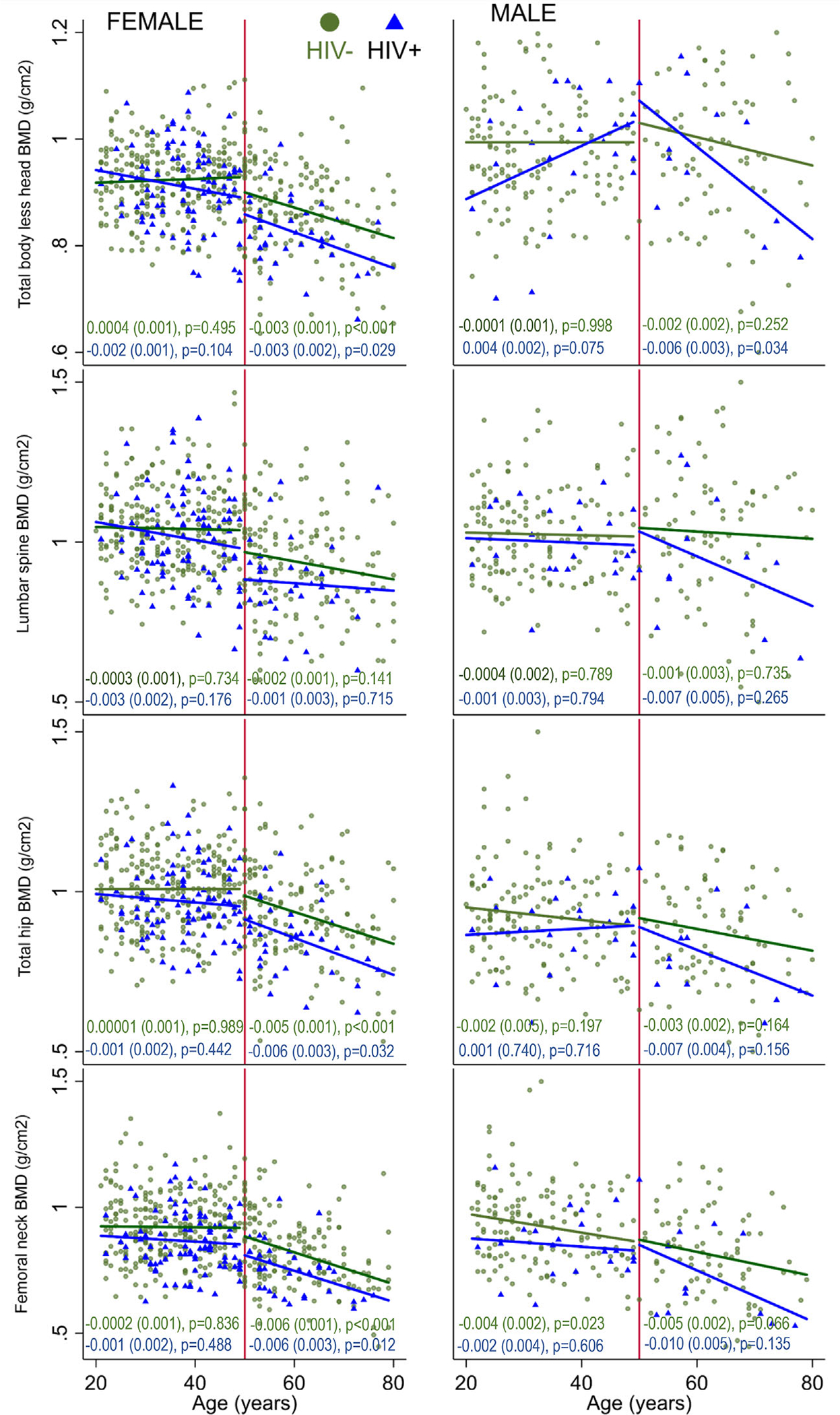
Unadjusted relationships between age and measures of BMD at four skeletal sites in females and males above and below the age of 50 years, living with and without HIV. Age against BMD is shown on scatter plots. Individuals with HIV are indicated by blue triangles, those without HIV by green circles. In women, 94 (27.2%) of those age <50 years and 35 (17.4%) of those ≥50 years were living with HIV. In men, 22 (13.5%) of those age <50 years and 12 (12.6%) of those ≥50 years were living with HIV. Piecewise regression lines are shown; β (standard error) represents g/cm^2^ per year of age. No evidence was detected for an interaction between age and HIV status on BMD outcomes in either the younger or older men or women in these unadjusted analyses. BMD = bone mineral density; HIV = human immunodeficiency virus.

In women, BMD measurements at all four skeletal sites were similar across the age range 20 to 50 years, both in those with and without HIV (Fig. 1). In women age ≥50 years, inverse associations were observed between age and BMD at the TB-LH, TH, and FN, but not at the LS, with no evidence that these inverse associations were stronger in those women living with HIV. In men above and below the age of 50 years there was little evidence of an association between age and BMD at any skeletal site (Fig. 1).

### Factors associated with BMD

In unadjusted analyses, older age, female sex, and HIV infection were all strongly associated with lower BMD at all four skeletal sites, and greater ALM/height^2^, grip strength, and gait speed were all strongly associated with higher BMD, again at all four skeletal sites. Greater fat mass was associated with greater LS, TH, and FN BMD, but lower TB-LH BMD. Physical activity was only weakly positively associated with TB-LH BMD (Supplemental Table S4). After initial adjustment for age and sex, the associations between HIV infection and lower BMD were still evident at all four skeletal sites, whereas the associations between greater ALM/height^2^ and grip strength both remained strongly associated with higher BMD (at all sites), as was fat mass. However, associations between both gait speed and physical activity and BMD were attenuated (Supplemental Table S5).

Multivariable models were then used to determine the independent associations between the exposures age, sex, HIV infection, grip strength, ALM/height^2^, gait speed, and fat mass, and BMD at each of the four skeletal sites. As expected, older age and female sex were consistently and independently associated with lower BMD at all four sites (Table 3). Of the three measures of sarcopenia, ALM/height^2^ showed the strongest independent relationships with FN and TH BMD, and to a lesser extent TB-LH, with no association with LS BMD. Grip strength was independently associated with BMD at the TH, LS, and TB-LH, but much less so at the FN. Gait speed was only weakly associated with FN BMD. In these multivariable analyses, fat mass was only associated with LS BMD, whereas HIV infection was only associated with FN BMD. Overall, the seven variables in our models explained almost one-half of the variance in TB-LH BMD, just over one-third at the two hip sites, and only 19% of the variance in LS BMD (VIF <3).

### Modification of the effect of age on BMD by HIV infection in women

Among the 335 women age <50 years with complete data for all variables, those with (*n* = 89) and without (*n* = 245) HIV had similar measures of TB-LH BMD (mean difference −0.013 [95% CI, −0.030, 0.0003] g/cm^2^), whereas in 187 women age ≥50 years, those with HIV (*n* = 33) had 0.045 [95% CI, 0.012–0.077] g/cm^2^ lower TB-LH BMD than those without HIV (*n* = 154) (interaction *p* = 0.027). Consistent but weaker patterns were seen at the TH, LS, and FN BMD.

## Discussion

We studied a rural-dwelling South African population with a high prevalence of HIV and obesity. Overall osteoporosis was common in those age ≥50 years and in women; with high reported ART use, HIV infection was strongly associated with osteoporosis at all skeletal sites; 37% of older women with HIV had femoral neck osteoporosis. In both men and women, HIV infection was associated with an approximately 0.5 SD lower femoral neck BMD. By contrast, overall, the numbers with probable sarcopenia were low, with no confirmed or severe sarcopenia identified.

Osteoporosis prevalence has not previously been estimated using data from a population-based study in Southern Africa. Two small cross-sectional studies from Cameroon and Nigeria, both using quantitative ultrasound to estimate bone density, reported a prevalence of osteoporosis of 17.9% and 18.2%, respectively, in women age ≥50 years or who were postmenopausal.^(32,33)^ A more recent cross-sectional study of 254 postmenopausal women living in a peri-urban setting in Kenya identified an osteoporosis prevalence of 26.4%; HIV was not examined.^(34)^ In contrast, the osteoporosis prevalence in US populations age ≥50 years is well known, affecting 3.9% and 15.8% of non-Hispanic white men and women, and 1.3% and 7.7% of non-Hispanic black men and women, respectively.^(35)^ The prevalence we report suggest osteoporosis is at least as common a problem in a rural South African population as it is in the US. Yet none of the women we studied reported use of any osteoporosis medications, including hormone replacement therapy, to ameliorate postmenopausal bone loss. Parity was high in this rural South African population. It is unclear whether short-term losses in bone density during pregnancy and lactation fully recover in this population, as they are thought to in high-income settings.^(36)^

The HIV-associated bone deficits identified with may in part be explained by the types of ART regimes used. Of those with HIV, 88% were established on ART. Although we lacked data on ART type and duration, at the time of this study the recommended first-line regime for adults in South Africa was (and still is) tenofovir disoproxil fumarate (TDF), lamivudine (3TC), and efavirenz (EFV), as a fixed dose combination.^(29)^ TDF in particular is known to cause 1% to 3% greater BMD loss in the first 2 years of therapy,^(37)^ by affecting renal tubular function leading to hyperphosphaturia and reduced skeletal mineralization.^(8)^

As expected, we saw inverse associations between age and BMD in women age ≥50 years, although not in the spine, likely due to degenerative changes artifactually increasing BMD as measured by DXA. Although we lacked accurate menopausal status data, we identified an age (above/below 50 years)*HIV interaction on TB-LH BMD in women, suggesting greater BMD losses may be experienced by postmenopausal women living with HIV than in those who are uninfected. This is consistent with findings from a small longitudinal study of postmenopausal US Hispanic and African-American women that showed greater bone loss in women living with HIV.^(6)^ To our knowledge ours are the first data to suggest this in an African population and warrant further longitudinal study, given that routine bone health assessment in perimenopausal women attending HIV clinics is not currently the standard of care in South Africa.

Our findings suggest either that, unlike osteoporosis, sarcopenia is not common in this population, or that use of the European thresholds, particularly for grip strength, which defines probable sarcopenia, is nondifferentiating, in which case further work is needed to identify context-specific sarcopenia definitions. A recent meta-analysis identified a 30.3% prevalence of low ALM, and 4.5% prevalence of sarcopenia, based on 2010 EWGSOP guidelines,^(38)^ in adults living with HIV, although only three of 41 evaluated studies were conducted in Africa; none used the newer EWGSOP2 definition.^(12,39)^ In the meta-analysis higher BMI attenuated HIV-associated deficits in lean mass, which may explain our findings in this obese South African population. Although the prevalence of osteoporosis was high and sarcopenia was uncommon, the three muscle measures of strength, lean mass, and gait speed were associated with BMD at different skeletal sites. Our multivariable models showed clear evidence for independent associations between lean mass index (ALM/height^2^) and hip BMD measures, between muscle strength and all BMD measures except FN BMD, and between gait speed and FN BMD. All three components of sarcopenia were independently associated with hip BMD, although ALM/height^2^ was the strongest independent predictor, suggesting each individual muscle measure may be influencing hip strength by independent means. Muscle and bone function are tightly linked; interestingly two small randomized trials in Iran and Malawi have recently reported beneficial effects of muscle training on BMD in adults living with HIV, potentially suggesting an intervention that may help, at least in part, ameliorate the negative effects of HIV on bone health.^(40,41)^ There is currently no consensus agreement over the definition of sarcopenia in male and female African populations.^(42,43)^

The prevalence of overweight and obesity in this rural community was striking and of public health concern, affecting 73% of women and 43% of men. The most recent national data from the South African Demographic and Health Survey (SADHS) in 2016 reported a prevalence of overweight and/or obesity of 66.1% and 26.1% in non-urban-dwelling women and men age ≥15 years, respectively.^(44)^ These estimates had increased from 2003 when SADHS reported a prevalence of 49.6% and 27.8%, respectively.^(45)^ Fat mass, particularly visceral fat, is thought to exert negative effects on bone quality, and hence obesity is recognized as an independent risk factor for fracture, over and above BMD.^(46)^ Hence the combination seen here, of low BMD and very high rates of obesity, raises serious concerns for future fragility fracture incidence.

We used NHANES III reference data from a white population in North America to derive BMD *Z*-scores and *T*-scores in this black South African population,^(21)^ as recommended by the ISCD in the absence of region-specific reference data.^(22)^ If these reference data were a good fit for this population, then those without HIV should have a mean *Z*-score close to 0 and SD close to 1. However, this was not seen, suggesting NHANES III data are not an ideal reference population for use in rural South Africa. This is unsurprising given evidence that North American and Southern African populations have marked differences in both bone density and geometry.^(47,48)^ Although studies are few, data suggest black South African women living in Johannesburg, have on average, shorter, wider femoral necks, with smaller neck-shaft angles and greater cortical thickness, compared with white South African women, which may explain the higher observed TH and FN *Z*-scores observed in this study.^(48)^ Unfortunately, our study population had too few men and women aged 20–29.9 years who did not have HIV, to robustly derive a reference mean and SD to generate *Z*-scores in the older individuals. However, the data we present were sampled to be representative of a rural South African community, and as such, it is now possible to use these data together with future population samples, to derive the first BMD reference data for South Africa.

### Strengths and limitations

Our study is the largest study to date of osteoporosis and sarcopenia conducted in community-based adults in Southern Africa.^(7,9)^ Limitations include the recruitment of fewer males than females, and hence male-specific CIs were wider and conclusions less certain. Although missing data were few, because 17% of the study sample were recruited through word-of-mouth (albeit from the same source population) rather than by household sampling, this may limit generalizability. Gait speed was measured based on fast walking rather than normal walking, which explains the relatively high gait speeds measured in this population, compared with others.^(49)^ We unfortunately lacked data on ART regimes, HIV infection duration and severity, and menopausal age. Because we were only able to study those who were alive and healthy enough to attend the research clinic, we may have underestimated the bone and muscle deficits in the population living (and dying) with HIV in this context. Given the widespread use of hairpieces in the female population in South Africa, it was important to use total body-less-head (TB-LH) estimates of body composition variables to avoid artifacts; however, NHANES normative data needed to calculate TB-LH *Z*-scores were only available for those age <70 years.^(21)^

## Conclusions

In rural South Africa, osteoporosis is common, affecting 19.4% of women and 8.4% of men aged over 50 years. HIV-associated bone deficits, particularly at the FN, raise concerns regarding future hip fracture risk.^(4)^ Further research is needed to understand the effect of HIV on menopausal bone losses in women living in Southern Africa. Although sarcopenia was uncommon, consensus on definitions specific to African populations is lacking. Our findings suggest that HIV clinics in South Africa should consider routine bone health assessment, particularly in older women; quantification of osteoporotic fracture risk is now possible with the recent launch of the new South African Fracture Risk Assessment Tool (FRAX).^(50)^ However, because osteoporosis medications are still not on the South African Essential Drugs List, and even among the private sector osteoporosis is still not considered a Prescribed Medical Benefit (PMB), osteoporosis treatment is likely to be remain limited until health policies change.^(51)^ Further follow-up of this population is warranted to determine fracture incidence and the extent to which BMD and HIV infection explain fracture risk.

## Supplementary Material

Supplementary Material

## Figures and Tables

**Table 1.** Characteristics of the Rural South African Studv PoDulation, Stratified bv Sex and HIV Status

Characteristic	*n*	Men				Women				*p* ^b^
		All (*n* = 258)	HIV+ (*n* = 34)	HIV− (*n* = 224)	*p* ^a^	All (*n* = 547)	HIV+ (*n* = 129)	HIV− (*n* = 418)	*p* ^a^	

Demographic and clinical variables										
Age (years), mean ± SD	805	44.1 ± 16.1	46.2 ± 14.3	43.8 ± 16.3	0.422	44.8 ± 14.2	44.1 ± 11.8	45.0 ± 14.9	0.55	0.537
SES quartile, *n* (%)	665				0.705				0.926	0.011
Q1 (lowest)		7 (3.5)	2 (6.1)	5 (3.1)		13 (2.8)	4 (3.2)	9 (2.6)		
Q2		38 (19.4)	7 (21.2)	31 (19.1)		55 (11.7)	13 (10.6)	42 (12.1)		
Q3		147 (75)	24 (72.7)	122 (75.3)		372 (79.3)	98 (79.7)	274 (28.3)		
Q4 (highest)		4 (2.0)	0	4 (2.5)		29 (6.2)	8 (6.5)	21 (6.1)		
Smoking tobacco, *n* (%)	773				0.022				0.341	<0.001
Never		159 (63.6)	15 (44.1)	144 (66.7)		516 (98.7)	126 (97.7)	390 (98.9)		
Ex-smoker		69 (27.6)	16 (47.1)	53 (24.5)		5 (0.9)	2 (1.6)	3 (0.8)		
Current		22 (8.8)	3 (8.8)	19 (8.8)		2 (0.4)	1 (0.7)	1 (0.3)		
Alcohol consumption, *n* (%)	329				0.536				0.057	<0.001
Never		27 (30.1)	4 (30.8)	23 (28.8)		204 (87.8)	39 (76.5)	165 (89.2)		
Previous		19 (19.5)	1 (7.7)	18 (22.5)		12 (4.1)	4 (7.8)	8 (4.3)		
Current		47 (50.4)	8 (61.5)	39 (48.8)		20 (8.1)	8 (15.7)	12 (6.5)		
Physical activity (hours/week), median (IQR)	269	3.5 (1.2–7.0)	3.0 (2.5–3.5)	3.5 (1.2–9.0)	0.423	1.8 (1.0–3.5)	2.5 (0.8–5.3)	1.8 (1.0–3.5)	0.512	0.015
Established on ART, *n* (%)	163	31 (91.2)	31 (91.2)	–	–	113 (87.6)	113 (87.6)	–	–	0.561
Anthropometry										
Height (cm), mean ± SD	805	172.6 ± 6.8	172.6 ± 6.2	172.6 ± 6.9	0.956	161.5 ± 6.0	161.5 ± 6.3	161.5 ± 5.9	0.9	<0.001
Weight (kg), mean ± SD	805	75.1 ± 17.3	67.8 ± 16.1	76.2 ± 17.2	0.008	77.9 ± 17.4	72.1 ± 15.4	79.7 ± 17.5	<0.001	0.014
BMI, *n* (%)	805				0.029				<0.001	<0.001
Underweight		13 (5.0)	3 (8.8)	10 (4.5)		9 (1.7)	5 (3.9)	4 (1.0)		
Normal		134 (51.9)	24 (70.6)	110 (49.1)		138 (25.2)	40 (31.0)	98 (23.4)		
Overweight		71 (27.5)	5 (14.7)	66 (29.5)		151 (27.6)	44 (34.1)	107 (25.6)		
Obese		40 (15.5)	2 (5.9)	38 (17.0)		249 (45.5)	40 (31.0)	209 (50.0)		

Minimum and maximum age (years) by sex and HIV status were as follows: female HIV+ (22–76), HIV− (21–79); male HIV+ (22–79), HIV− (21–79).

ART = anti-retroviral therapy; BMI = body mass index; HIV = human immunodeficiency virus; IQR = interquartile range; SD = Standard deviation; SES = socioeconomic status.

a HIV+ versus HIV−.

b AII males versus all females.

**Table 2.** Comparison of Bone and Muscle Measures, and the Prevalence of Osteoporosis and Sarcopenia, by Sex and HIV Status

Para meter	Men				Women				*p* ^b^
	All (*n* = 258)	HIV + (*n* = 34)	HIV − (*n* = 224)	*p* ^a^	All (*n* = 547)	HIV + (*n* = 129)	HIV − (*n* = 418)	*p* ^a^	

BMD (g/cm^2^), mean ± SD (*n* = 805)									
FN	0.955 ± 0.161	0.893 ± 0.151	0.965 ± 0.160	0.015	0.870 ± 0.142	0.834 ± 0.124	0.882 ± 0.146	0.001	<0.001
TH	1.077 ± 0.149	1.033 ± 0.123	1.084 ± 0.152	0.064	0.969 ± 0.137	0.938 ± 0.128	0.979 ± 0.139	0.003	<0.001
LS	1.069 ± 0.157	1.033 ± 0.132	1.075 ± 0.160	0.142	0.996 ± 0.155	0.974 ± 0.154	1.003 ± 0.155	0.063	<0.001
TB-LH	1.046 ± 0.094	1.032 ± 0.084	1.048 ± 0.096	0.36	0.898 ± 0.081	0.887 ± 0.082	0.902 ± 0.080	0.069	<0.001
BMD *Z*-score, mean ± SD (*n* = 805)									
FN	0.68 ± 1.20	0.25 ± 1.12	0.74 ± 1.21	0.027	0.54 ± 1.12	0.18 ± 0.92	0.65 ± 1.15	<0.001	0.104
TH	0.37 ± 1.11	0.06 ± 0.85	0.41 ± 1.13	0.086	0.37 ± 1.06	0.07 ± 0.91	0.45 ± 1.09	<0.001	0.994
LS	0.12 ± 1.19	−0.11 ± 0.95	0.16 ± 1.22	0.218	−0.24 ± 1.14	−0.47 ± 1.12	−0.16 ± 1.14	0.007	<0.001
TB-LH^c^	−0.59 ± 0.97	−0.69 ± 0.89	−0.58 ± 0.99	0.538	−0.86 ± 0.90	−1.04 ± 0.84	−0.80 ± 0.91	0.008	<0.001
BMD *T*-score, *n* (%) (*n* = 296 aged ≥50 years)									
FN				0.19				0.003	<0.001
≥ −1	61 (64.2)	5 (41.7)	56 (67.5)		68 (33.8)	5 (14.3)	63 (38.0)		
> −2.5 and < −1	30 (31.6)	6 (50.0)	24 (28.9)		94 (46.8)	17 (48.6)	77 (46.4)		
≤ −2.5	4 (4.2)	1 (8.3)	3 (3.6)		39(19.4)	13 (37.1)	26 (15.7)		
TH				0.143				0.04	0.772
≥ −1	67 (70.5)	6 (50.0)	61 (73.5)		138 (68.7)	18 (51.4)	120 (72.3)		
> −2.5 and < −1	25 (26.3)	6 (50.0)	19 (22.9)		53 (26.4)	14 (40.0)	39 (23.5)		
≤ −2.5	3 (3.2)	0	3 (3.6)		10 (5.0)	3 (8.6)	7 (4.2)		
LS				0.415				0.005	<0.001
≥ −1	72 (75.8)	9 (75.0)	63 (75.9)		81 (40.3)	6 (17.1)	75 (45.2)		
> −2.5 and < −1	15 (15.8)	1 (8.3)	14 (16.9)		83 (41.3)	22 (62.9)	61 (36.8)		
≤ −2.5	8 (8.4)	2 (16.7)	6 (7.2)		37 (18.4)	7 (20.0)	30 (18.1)		
TB-LH				0.717				0.007	<0.001
≥ −1	62 (65.3)	7 (58.3)	55 (66.3)		69 (34.4)	4 (11.4)	65 (39.2)		
> −2.5 and < −1	29 (30.5)	5 (41.7)	24 (28.9)		99 (49.3)	24 (68.6)	75 (45.2)		
≤ −2.5	4 (4.2)	0	4 (4.8)		33 (16.4)	7 (20.0)	26 (15.7)		
Muscle and fat assessments (*n* = 805)				0.843				0.644	<0.001
Grip strength (kg), mean ± SD	40.1 ± 11.2	39.7 ± 8.2	40.1 ± 11.6		27.8 ± 7.2	28.0 ± 7.2	27.7 ± 7.2		
Low grip strength, *n* (%)^d^	30 (11.7)	4 (11.8)	26 (11.7)	0.986	24 (4.4)	3 (2.3)	21 (5.1)	0.227	<0.001
Galt speed (m/s), mean ± SD^e^	1.92 ± 0.29	1.92 ± 0.28	1.92 ± 0.29	0.959	1.65 ± 0.22	1.65 ± 0.20	1.65 ± 0.23^f^	0.976	<0.001
Weight (kg), mean ± SD	75.1 ± 17.3	67.8 ± 16.1	76.2 ± 17.2	0.008	77.9 ± 17.4	72.1 ± 15.4	79.7 ± 17.5	<0.001	0.014
TB-LH fat mass (kg), mean ± SD	16.9 ± 8.86	12.7 ± 8.44	17.6 ± 8.76	0.002	30.4 ± 11.04	26.7 ± 9.78	31.6 ± 11.17	<0.001	<0.001
TB-LH lean mass (kg), mean ± SD^g^	52.8 ± 9.1	49.8 ± 8.6	53.2 ± 9.1	0.044	42.6 ± 6.8	40.7 ± 6.3	43.2 ± 6.86	<0.001	<0.001
ALM (kg), mean ± SD	26.1 ± 4.9	24.3 ± 4.3	26.3 ± 4.9	0.026	20.5 ± 3.7	19.4 ± 3.4	20.9 ± 3.7	<0.001	<0.001
ALM/helght^2^, mean ± SD	8.8 ± 1.4	8.2 ± 1.3	8.9 ± 1.4	0.005	7.9 ± 1.3	7.4 ± 1.1	8.0 ± 1.3	<0.001	<0.001
Low ALM/height^2^, *n* (%)	19 (7.4)	4 (11.8)	15 (6.7)	0.291	5 (0.91)	2 (1.6)	3 (0.72)	0.337	<0.001
Sarcopenia, *n* (%)				0.979				0.191	0.001
No sarcopenia	228 (88.4)	30 (88.2)	198 (88.4)		523 (95.6)	126 (97.7)	397 (95.0)		
Sarcopenia probable^h^	30 (11.6)	4 (11.8)	26 (11.6)		24 (4.4)	3 (2.3)	21 (5.0)		

ALM = appendicular lean mass; BMD = bone mineral density; FN = femoral neck; HIV = human immunodeficiency virus; LS = lumbar spine; SD = Standard deviation; TB-LH = total body less head; TH = total hip.

a HIV+ versus HIV−.

b AII males versus all females.

c *n* = 758 as NHANES normative data needed to calculate TB-LH Z-scores were only available for those aged <70 years.

d Grip strength missing in four individuals.

e Gait speed missing in 36 individuals.

f Only one HIV− female had a low gait speed (≤0.8 m/s).

g Lean mass measured as fat-free soft tissue mass.

h There were no cases of confirmed or severe sarcopenia.

**Table 3.** Multivariable Analysis to Determine Risk Factors Independently Associated With BMD for Each of the Four Skeletal Sites

Parameter	β coefficient	95% CI	*p*	*r* ^2^

FN BMD (g/cm^2^)				0.37
Age (decade)	−0.035	(−0.042, −0.028)	<0.001	
Sex (female)	−0.077	(−0.113, −0.040)	<0.001	
HIV infection	−0.024	(−0.046, −0.002)	0.031	
Grip strength	0.010	(−0.001, 0.021)	0.065	
ALM/height^2^	0.047	(0.023, 0.070)	<0.001	
Gait speed	0.013	(0.001, 0.024)	0.031	
Fat mass	0.016	(−0.011, 0.043)	0.238	
TH BMD (g/cm^2^)				0.36
Age (decade)	−0.024	(−0.031, −0.017)	<0.001	
Sex (female)	−0.096	(−0.133, −0.060)	<0.001	
HIV infection	−0.015	(−0.036, 0.007)	0.192	
Grip strength	0.014	(0.003, 0.025)	0.012	
ALM/height^2^	0.045	(0.022, 0.069)	<0.001	
Gait speed	0.010	(−0.002, 0.021)	0.093	
Fat mass	0.016	(−0.011, 0.042)	0.253	
LS BMD (g/cm^2^)				0.19
Age (decade)	−0.025	(−0.033–0.016)	<0.001	
Sex (female)	−0.103	(−0.146, −0.060)	<0.001	
HIV infection	−0.009	(−0.035, 0.017)	0.511	
Grip strength	0.016	(0.003, 0.029)	0.015	
ALM/height^2^	0.009	(−0.019, 0.036)	0.528	
Gait speed	−0.003	(−0.017, 0.010)	0.646	
Fat mass	0.046	(0.014, 0.078)	0.005	
TB-LH BMD (g/cm^2^)				0.48
Age (decade)	−0.010	(−0.015, −0.006)	<0.001	
Sex (female)	−0.127	(−0.151, −0.103)	<0.001	
HIV infection	−0.004	(−0.018, 0.010)	0.596	
Grip strength	0.012	(0.005, 0.019)	0.001	
ALM/height^2^	0.022	(0.006, 0.037)	0.005	
Gait speed	0.005	(−0.002, 0.013)	0.161	
Fat mass	0.002	(−0.016, 0.019)	0.863	

β coefficient represents the SD increase in BMD per SD increase in continuous exposures, or decade increase in age, being female versus male, or having HIV+ versus HIV−. Each β coefficient is adjusted for the other six variables in each model. *r*^2^ indicates the proportion of variance in the BMD outcome explained by the seven variables in the model.

ALM = appendicular lean mass; BMD = bone mineral density; CI = confidence interval; FN = femoral neck; HIV = human immunodeficiency virus; LS = lumbar spine; TB-LH = total body less head; TH = total hip.

## Data Availability

Data sharing: The data that support the findings of this study are available on request from the senior authors. The data are not publicly available due to privacy/ ethical restrictions.

## References

[1] Aboderin IA, Beard JR. Older people’s health in sub-Saharan Africa. Lancet. 2015;385(9968):e9–e11. 25468150 10.1016/S0140-6736(14)61602-0

[2] Clynes MA, Gregson CL, Bruyère O, Cooper C, Dennison EM. Osteosarcopenia: where osteoporosis and sarcopenia collide. Rheumatology (Oxford). 2021;60(2):529–537. 33276373 10.1093/rheumatology/keaa755

[3] Conradie M, Conradie MM, Scher AT, Kidd M, Hough S. Vertebral fracture prevalence in black and white South African women. Arch Osteoporos. 2015;10(1):1. 10.1007/s11657-015-0203-x 25675880

[4] Dela SS, Paruk F, Brown SL, Ethnic and gender-specific incidence rates for hip fractures in South Africa: a multi-centre study. Bone. 2020;133:115253. 31987987 10.1016/j.bone.2020.115253

[5] Mlangeni L, Makola L, Naidoo I, Factors associated with physical activity in South Africa: evidence from a national population based survey. Open Public Health J. 2018;11:516–525.

[6] Yin MT, Zhang CA, McMahon DJ, Higher rates of bone loss in postmenopausal HIV-infected women: a longitudinal study. J Clin Endocrinol Metab. 2012;97(2):554–562. 22090266 10.1210/jc.2011-2197 PMC3275353

[7] Hamill MM, Pettifor JM, Ward KA, Norris SA, Prentice A. Changes in bone mineral density, body composition, vitamin D status, and mineral metabolism in urban HIV-positive south African women over 12 months. J Bone Miner Res. 2017;32(8):1615–1624. 28370356 10.1002/jbmr.3147 PMC5753880

[8] Delpino MV, Quarleri J. Influence of HIV infection and antiretroviral therapy on bone homeostasis. Front Endocrinol. 2020;11:502. 10.3389/fendo.2020.00502 PMC7493215 32982960

[9] Dave JA, Cohen K, Micklesfield LK, Maartens G, Levitt NS. Antiretroviral therapy, especially Efavirenz, is associated with low bone mineral density in HIV-infected south Africans. PLoS One. 2015;10(12):e0144286. 26633015 10.1371/journal.pone.0144286 PMC4669137

[10] Hawkins KL, Brown TT, Margolick JB, Erlandson KM. Geriatric syndromes: new frontiers in HIV and sarcopenia. AIDS (London, England). 2017;31(Suppl 2):S137–S146. 28471944 10.1097/QAD.0000000000001444 PMC5693390

[11] Oliveira VHF, Borsari AL, Webel AR, Erlandson KM, Deminice R. Sarcopenia in people living with the human immunodeficiency virus: a systematic review and meta-analysis. Eur J Clin Nutr. 2020;74(7):1009–1021. 32341489 10.1038/s41430-020-0637-0

[12] Cruz-Jentoft AJ, Bahat G, Bauer J, Sarcopenia: revised European consensus on definition and diagnosis. Age Ageing. 2019;48(1):16–31. 30312372 10.1093/ageing/afy169 PMC6322506

[13] Jaff NG, Norris SA, Snyman T, Toman M, Crowther NJ. Body composition in the Study of Women Entering and in Endocrine Transition (SWEET): a perspective of African women who have a high prevalence of obesity and HIV infection. Metabolism. 2015;64(9):1031–1041. 26031506 10.1016/j.metabol.2015.05.009

[14] Mhariwa PC, Myezwa H, Galantino ML, Maleka D. The relationship between lower limb muscle strength and lower extremity function in HIV disease. S Afr J Physiother. 2017;73(1):360. 30135905 10.4102/sajp.v73i1.360 PMC6093131

[15] Kruger HS, Havemann-Nel L, Ravyse C, Moss SJ, Tieland M. Physical activity energy expenditure and sarcopenia in black south African urban women. J Phys Act Health. 2016;13(3):296–302. 26182196 10.1123/jpah.2015-0078

[16] Simbayi L, Zuma K, Moyo S, South African National HIV Prevalence, Incidence, Behaviour and Communication Survey, 2017. The Human Sciences Research Council (HSRC); 2019. Available from: https://www.hsrcpress.ac.za/books/south-african-national-hiv-prevalence-incidence-behaviour-and-communication-survey-2017.

[17] Kahn K, Collinson MA, Gomez-Olive FX, Profile: Agincourt health and socio-demographic surveillance system. Int J Epidemiol. 2012;41(4):988–1001. 22933647 10.1093/ije/dys115 PMC3429877

[18] Kahn K, Tollman SM, Collinson MA, Research into health, population and social transitions in rural South Africa: data and methods of the Agincourt health and demographic surveillance system. Scand J Public Health Suppl. 2007;69:8–20. 17676498 10.1080/14034950701505031 PMC2826136

[19] MRC/Wits Agincourt Unit Agincourt Study Area Map. 2021. Available at: https://www.agincourt.co.za/?page_id=1896.

[20] Kalyesubula R, Fabian J, Nakanga W, How to estimate glomerular filtration rate in sub-Saharan Africa: design and methods of the African Research into Kidney Diseases (ARK) study. BMC Nephrol. 2020;21(1):20. 31941441 10.1186/s12882-020-1688-0 PMC6964098

[21] Looker AC, Borrud LG, Hughes JP, Fan B, Shepherd JA, Melton LJ 3rd. Lumbar spine and proximal femur bone mineral density, bone mineral content, and bone area: United States, 2005–2008. Vital Health Stat 11. 2012;251:1–132. 24261130

[22] International Society for Clinical Densitometry. ISCD Offical Positions Adult. 2019. Available from: https://iscd.org/learn/official-positions/adult-positions/.

[23] Kim M, Shinkai S. Prevalence of muscle weakness based on different diagnostic criteria in community-dwelling older adults: a comparison of grip strength dynamometers. Geriatr Gerontol Int. 2017;17(11):2089–2095. 28517036 10.1111/ggi.13027

[24] Bohannon RW. Comfortable and maximum walking speed of adults aged 20–79 years: reference values and determinants. Age Ageing. 1997;26(1):15–19. 10.1093/ageing/26.1.15 9143432

[25] World Health Organization (WHO). The WHO STEPwise Approach to Surveillance of Noncommunicable Diseases (STEPS). Geneva: WHO; 2003. Available from: https://www.who.int/ncd_surveillance/en/steps_framework_dec03.pdf.

[26] Kabudula CW, Houle B, Collinson MA, Kahn K, Tollman S, Clark S. Assessing changes in household socioeconomic status in rural South Africa, 2001–2013: A distributional analysis using household asset indicators. Soc Indicat Res. 2017;133(3):1047–1073. 10.1007/s11205-016-1397-z PMC5579134 28931968

[27] Cleland CL, Hunter RF, Kee F, Cupples ME, Sallis JF, Tully MA. Validity of the Global Physical Activity Questionnaire (GPAQ) in assessing levels and change in moderate-vigorous physical activity and sedentary behaviour. BMC Public Health. 2014;14(1):1255. 25492375 10.1186/1471-2458-14-1255 PMC4295403

[28] Bull FC, Maslin TS, Armstrong T. Global Physical Activity Questionnaire (GPAQ): nine-country reliability and validity study. J Phys Act Health. 2009;6(6):790–804. 20101923 10.1123/jpah.6.6.790

[29] National Department of Health Republic of South Africa National Consolidated Guidelines for the Prevention of Mother-To-Child Transmission of HIV (PMTCT) and the Management of HIV In Children, Adolescents and Adults. 2015. https://sahivsoc.org/Files/ART20Guidelines2015052015.pdf.

[30] Harris PA, Taylor R, Minor BL, The REDCap consortium: building an international community of software platform partners. J Biomed Inform. 2019;95:103208. 31078660 10.1016/j.jbi.2019.103208 PMC7254481

[31] Harris PA, Taylor R, Thielke R, Payne J, Gonzalez N, Conde JG. Research electronic data capture (REDCap)-a metadata-driven methodology and workflow process for providing translational research informatics support. J Biomed Inform. 2009;42(2):377–381. 18929686 10.1016/j.jbi.2008.08.010 PMC2700030

[32] Singwe-Ngandeu M, Nko’o Amvene S. [Bone mineral density in Cameroon women in Yaounde: an echographic study]. Mali Med. 2008;23(1):21–26. French. 19437809

[33] VanderJagt DJ, Bond B, Dulai R, Assessment of the bone status of Nigerian women by ultrasound and biochemical markers. Calcif Tissue Int. 2001;68(5):277–284. 11683534 10.1007/BF02390834

[34] Sitati FC, Gichangi P, Obimbo MM. Prevalence of osteoporosis and its associated factors among postmenopausal women in Kiambu County, Kenya: a household survey. Arch Osteoporos. 2020;15(1):31. 32112149 10.1007/s11657-020-0685-z

[35] Wright NC, Looker AC, Saag KG, The recent prevalence of osteoporosis and low bone mass in the United States based on bone mineral density at the femoral neck or lumbar spine. J Bone Miner Res. 2014;29(11):2520–2526. 24771492 10.1002/jbmr.2269 PMC4757905

[36] Watts NB, Binkley N, Owens CD, Bone mineral density changes associated with pregnancy, lactation, and medical treatments in premenopausal women and effects later in life. J Womens Health (Larchmt). 2021;30(10):1416–1430. 34435897 10.1089/jwh.2020.8989 PMC13174973

[37] Grant PM, Cotter AG. Tenofovir and bone health. Curr Opin HIV AIDS. 2016;11(3):326–332. 26859637 10.1097/COH.0000000000000248 PMC4844450

[38] Cruz-Jentoft AJ, Baeyens JP, M B Jr, Sarcopenia: European consensus on definition and diagnosis. Age Ageing. 2010;39(4):412–423. 20392703 10.1093/ageing/afq034 PMC2886201

[39] Guimarães NS, Raposo MA, Greco D, Tupinambas U, Premaor MO. People living with HIV, lean mass, and sarcopenia: a systematic review and meta-analysis. J Clin Densitom. 2021;S1094–6950(21):00022–00026. 10.1016/j.jocd.2021.03.004. 33836973

[40] Ghayomzadeh M, Earnest CP, Hackett D, Combination of resistance and aerobic exercise for six months improves bone mass and physical function in HIV infected individuals: a randomized controlled trial. Scand J Med Sci Sports. 2021;31(3):720–732. 33185897 10.1111/sms.13871

[41] Chisati EM, Constantinou D, Lampiao F. Effects of maximal strength training on bone mineral density in people living with HIV and receiving anti-retroviral therapy: a pilot study. BMC Sports Sci Med Rehabil. 2020;12:67. 33110607 10.1186/s13102-020-00216-6 PMC7585307

[42] Kruger HS, Micklesfield LK, Wright HH, Havemann-Nel L, Goedecke JH. Ethnic-specific cut-points for sarcopenia: evidence from black South African women. Eur J Clin Nutr. 2015;69(7):843–849. 25604775 10.1038/ejcn.2014.279

[43] Mendham AE, Lundin-Olsson L, Goedecke JH, Sarcopenic obesity in Africa: a call for diagnostic methods and appropriate interventions. Front Nutr. 2021;8:661170. 10.3389/fnut.2021.661170. 33937309 PMC8085278

[44] National Department of Health (NDoH), Statistics South Africa (Stats SA), South African Medical Research Council (SAMRC), and ICF. 2019. South Africa Demographic and Health Survey 2016. Pretoria, South Africa, and Rockville, Maryland, USA: NDoH, Stats SA, SAMRC, and ICF. Available at: https://dhsprogram.com/pubs/pdf/FR337/FR337.pdf

[45] Department of Health/South Africa, Medical Research Council/South Africa, ORC Macro. South Africa Demographic and Health Survey 2003. Pretoria, South Africa: Department of Health/South Africa, 2007.

[46] Shapses SA, Pop LC, Wang Y. Obesity is a concern for bone health with aging. Nutr Res. 2017;39:1–13. 28385284 10.1016/j.nutres.2016.12.010 PMC5385856

[47] Mukwasi C, Stranix Chibanda L, Banhwa J, Shepherd JA. US white and black women do not represent the bone mineral density of sub-Saharan black women. J Clin Densitom. 2015;18:525–532. 26073424 10.1016/j.jocd.2015.05.065

[48] Nelson DA, Pettifor JM, Barondess DA, Cody DD, Uusi-Rasi K, Beck TJ. Comparison of cross-sectional geometry of the proximal femur in white and black women from Detroit and Johannesburg. J Bone Miner Res. 2004;19(4):560–565. 15005842 10.1359/JBMR.040104

[49] Bohannon RW, Williams AA. Normal walking speed: a descriptive meta-analysis. Physiotherapy. 2011;97(3):182–189. 21820535 10.1016/j.physio.2010.12.004

[50] Johansson H, Dela SS, Cassim B, FRAX-based fracture probabilities in South Africa. Arch Osteoporos. 2021;16(1):51. 33649966 10.1007/s11657-021-00905-w PMC7921059

[51] Hough S, Brown S, Cassim B, Improved management of patients with osteoporosis. S Afr Med J. 2012;102(11 Pt 1):815. 10.7196/samj.6317 23281547

