# Supplementary material for: Osteoporosis, Rather Than Sarcopenia, Is the Predominant Musculoskeletal Disease in a Rural South African Community Where Human Immunodeficiency Virus Prevalence Is High: A Cross-Sectional Study
Source: J Bone Miner Res. Author manuscript; Available in PMC 2023 Apr 4. (PMC10071443; doi:10.1002/jbmr.4464)
Supplement: Supplementary Material [file NIHMS1881564-supplement-Supplementary_Material.docx]

**Supplementary materials**

**Osteoporosis, rather than sarcopenia, is the predominant musculoskeletal disease in a rural South African community where HIV prevalence is high: a cross-sectional study. Gregson et al.**

**Supplemental Figure 1**

**Flow diagram illustrating the three-phase process of participant recruitment.**


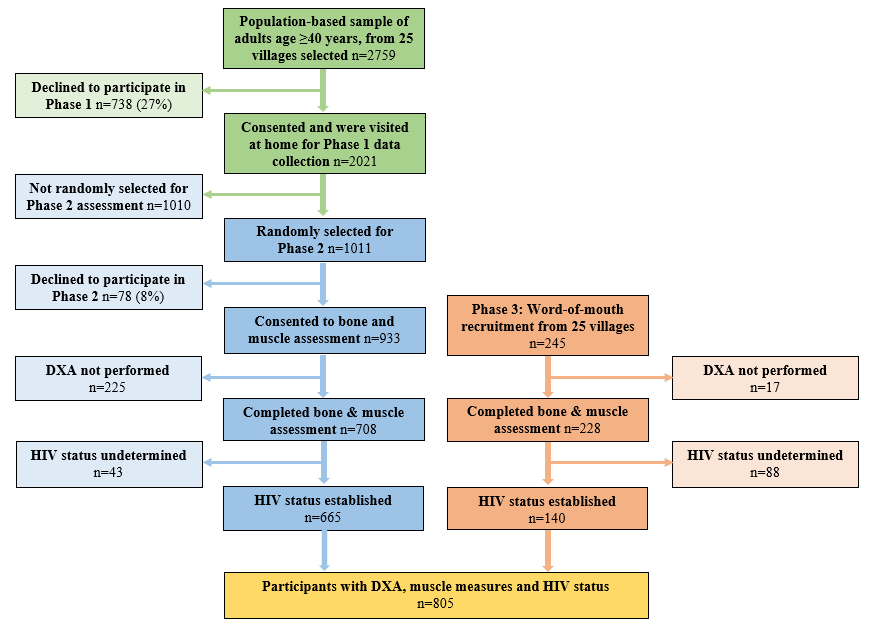


**Supplemental Table 1: Characteristics of those participants with and without complete data for DXA and muscle measures and HIV status**

|  | **Total**  **(n=2266)** | **Participants with**  **DXA data and HIV status established (n=805)** | **Invited but not participating (n=1461^a^)** | **p value** ^b^ |
| --- | --- | --- | --- | --- |
| **Age**, mean (SD) | 39.2 (14.4) | 43.8 (14.9) | 36.7 (13.6) | <0.001 |
| **Sex**, n (%)  Male  Female | 916 (40.4)  1350 (59.6) | 258 (32.1)  547 (67.9) | 658 (45.0)  803 (55.0) | <0.001 |
| **BMI**, n (%)  Underweight  Normal  Overweight  Obese | 88 (3.9)  901 (39.8)  616 (27.2)  661 (29.2) | 22 (2.7)  272 (33.8)  222 (27.6)  289 (35.9) | 66 (4.5)  629 (43.1)  394 (27.0)  372 (25.4) | <0.001 |
| **SES**, n (%)  Q1 (lowest)  Q2  Q3  Q4 (highest)  Missing | 97 (4.8)  267 (13.2)  1507 (74.6)  150 (7.4)  245 | 24 (3.6)  103 (15.5)  481 (72.3)  57 (8.6)  140 | 73 (5.4)  164 (12.1)  1026 (75.7)  93 (6.9)  105 | 0.026 |
| **HIV**, n (%)  Uninfected  Infected  Missing | 1674 (75.6)  376 (18.3)  216 | 642 (79.8)  163 (20.2)  0 | 1032 (82.9)  213 (17.1)  216 | 0.073 |

SD: Standard Deviation. BMI: Body Mass Index. SES: Socio-economic status (Quartiles). DXA: Dual-energy X-ray Absorptiometry

^a^ 1461 included 1088 who were selected in Phase 1 but not in Phase 2, plus 373 who were invited in Phase 2 or 3, but who either did not have a DXA performed, or did not have their HIV status confirmed. ^b^ calculated excluding missing values.

**Supplemental Table 2: Characteristics of participants with and without gait-speed and grip strength recorded**

|  | **Total**  **(n=805)** | **With gait-speed and grip strength**  **(n=769)** | **Without gait speed and grip strength**  **(n=36)** |
| --- | --- | --- | --- |
| Sex, n (%)  Male  Female | 258 (32.1)  547 (67.9) | 248 (32.3)  521 (67.7) | 10 (27.8)  26 (72.2) |
| Age, mean (SD) | 44.6 (14.8) | 44 (14.6) | 54.1 (15.7) |
| HIV status, n (%)  Negative  Positive | 642 (79.7)  163 (20.3) | 613 (79.7)  156 (20.3) | 29 (80.6)  7 (19.4) |
| BMI, n (%)  Underweight  Normal  Overweight  Obese | 22 (2.7)  272 (33.8)  222 (27.6)  289 (35.9) | 22 (2.9)  264 (34.3)  205 (26.7)  278 (36.1) | 0  8 (22.2)  17 (47.2)  11 (30.6) |

SD: Standard Deviation. BMI: Body Mass Index.

**Supplemental Table 3: The associations between BMD T-Score and probable sarcopenia in men and women aged 50 years and older**

| **BMD T-score**  **(n=296)** | **No Sarcopenia (n=270)** | **Probable Sarcopenia (n=26)** | **Odds Ratio**  **(95% CI)** | **p value** |
| --- | --- | --- | --- | --- |
| TB-LH, n (%)  ≥ -1  <-1, > -2.5  ≤ -2.5 | 120 (44.4)  117 (43.3)  33 (44.5) | 11 (47.8)  11 (43.5)  4 (8.7) | ref  1.03 (0.43, 2.48)  1.32 (0.40, 4.42) | 0.650 |
| LS, n (%)  ≥ -1  <-1, > -2.5  ≤ -2.5 | 137 (50.7)  94 (34.8)  39 (14.4) | 16 (65.2)  4 (17.4)  6 (17.4) | ref  0.36 (0.18, 1.11)  1.31 (0.48, 3.57) | 0.590 |
| FN, n (%)  ≥ -1  <-1, > -2.5  ≤ -2.5 | 119 (44.1)  113 (41.9)  38 (14.1) | 10 (43.5)  11 (43.5)  5 (13.0) | ref  1.14 (0.47, 2.81)  1.55 (0.50, 4.83) | 0.438 |
| TH, n (%)  ≥ -1  <-1, > -2.5  ≤ -2.5 | 190 (70.4)  71 (26.3)  9 (3.3) | 15 (62.2)  7 (26.1)  4 (8.7) | ref  1.24 (0.49, 3.17)  5.60 (1.54, 20.3) | 0.009 |

TB-LH: Total-body less-head; LS: Lumbar spine; FN: Femoral neck; TH; Total hip; CI: Confidence Interval

**Supplemental Table 4: Unadjusted associations between exposures and BMD measured at all 4 skeletal sites**

| **Exposures** | **TB-LH BMD (*g/cm^2^)*** | **LS BMD (*g/cm^2^)*** | **TH BMD (*g/cm^2^)*** | **FN BMD (*g/cm^2^)*** |
| --- | --- | --- | --- | --- |
| Age (per decade) | -0.014 (-0.019, -0.009) ^a^ | -0.022 (-0.030, -0.015) ^a^ | -0.027 (-0.033, -0.020) ^a^ | -0.038 (-0.044, -0.031) ^a^ |
| Female sex | -0.147 (-0.160, -0.134) ^a^ | -0.073 (-0.095, -0.048) ^a^ | -0.108 (-0.129, -0.087) ^a^ | -0.089 (-0.111, -0.066) ^a^ |
| HIV infection | -0.035 (-0.054, -0.017) ^a^ | -0.042 (-0.069, -0.015) ^a^ | -0.058 (-0.083, -0.032) ^a^ | -0.067 (-0.094, -0.041) ^a^ |
| Height (per SD) | 0.064 (0.058, 0.071) ^a^ | 0.053 (0.043, 0.064) ^a^ | 0.054 (0.044, 0.064) ^a^ | 0.055 (0.045, 0.065) ^a^ |
| Fat mass (per SD) | -0.018 (-0.026, -0.010) ^a^ | 0.022 (0.011, 0.033) ^a^ | 0.018 (0.008, 0.029) ^a^ | 0.023 (0.012, 0.033) ^a^ |
| ASM/height^2^ (per SD) | 0.024 (0.016, 0.032) ^a^ | 0.047 (0.036, 0.058) ^a^ | 0.059 (0.049, 0.068) ^a^ | 0.059 (0.049, 0.069) ^a^ |
| Grip strength (per SD) | 0.054 (0.047, 0.060) ^a^ | 0.041 (0.030, 0.052) ^a^ | 0.055 (0.045, 0.065) ^a^ | 0.051 (0.041, 0.061) ^a^ |
| Gait speed (per SD) | 0.043 (0.036 0.050) ^a^ | 0.027 (0.015, 0.038) ^a^ | 0.045 (0.035, 0.055) ^a^ | 0.050 (0.039, 0.060) ^a^ |
| Physical activity (per SD) | 0.014 (0.001, 0.027) ^b^ | 0.013 (-0.005, 0.032) | 0.001 (-0.016, 0.019) | 0.002 (-0.015, 0.019) |

N=805. Beta coefficient [95% CI] shown. ^a^p<0.001, ^b^p<0.05

SD: standard deviation; TB-LH: Total-body less-head; LS: Lumbar spine; FN: Femoral neck; TH; Total hip

Per SD: here beta represents the BMD increase per SD increase in exposure

**Supplemental Table 5: Age and sex adjusted associations between exposures and BMD measured at all 4 skeletal sites**

| **Exposures** | **TB-LH BMD (*g/cm^2^)*** | **LS BMD (*g/cm^2^)*** | **TH BMD (*g/cm^2^)*** | **FN BMD (*g/cm^2^)*** |
| --- | --- | --- | --- | --- |
| HIV infection | -0.016 (-0.030, -0.001)^b^ | -0.032 (-0.059, -0.005)^b^ | -0.045 (-0.068, -0.021)^a^ | -0.055 (-0.079, -0.031)^a^ |
| Height (per SD) | 0.033 (0.026, 0.040)^a^ | 0.049 (0.035, 0.062)^a^ | 0.030 (0.018, 0.043)^a^ | 0.039 (0.027, 0.052)^a^ |
| Fat mass (per SD) | 0.027 (0.020, 0.034)^a^ | 0.059 (0.047, 0.071)^a^ | 0.068 (0.058, 0.078)^a^ | 0.070 (0.059, 0.080)^a^ |
| ASM/height^2^ (per SD) | 0.025 (0.019, 0.031)^a^ | 0.049 (0.039, 0.059)^a^ | 0.061 (0.052, 0.069)^a^ | 0.063 (0.054, 0.071)^a^ |
| Grip strength (per SD) | 0.019 (0.012, 0.026)^a^ | 0.026 (0.013, 0.039)^a^ | 0.031 (0.019, 0.042)^a^ | 0.028 (0.016, 0.039)^a^ |
| Gait speed (per SD) | 0.006 (-0.001, 0.014) | -0.005 (-0.019, 0.009) | 0.008 (-0.004, 0.021) | 0.010 (-0.002, 0.023) |
| Physical activity (per SD) | 0.0001 (-0.010, 0.011) | 0.005 (-0.013, 0.022) | -0.009 (-0.025, 0.007) | -0.008 (-0.024, 0.008) |

N=805. Beta coefficient [95% CI] shown. ^a^p<0.001, ^b^p<0.05

SD: standard deviation; TB-LH: Total-body less-head; LS: Lumbar spine; FN: Femoral neck; TH; Total hip

Per SD: here beta represents the BMD increase per SD increase in exposure
